# Genetic Improvement of Wheat for Drought Tolerance: Progress, Challenges and Opportunities

**DOI:** 10.3390/plants11101331

**Published:** 2022-05-18

**Authors:** Theresa Bapela, Hussein Shimelis, Toi John Tsilo, Isack Mathew

**Affiliations:** 1 African Centre for Crop Improvement, University of Kwa-Zulu Natal, Private Bag X01, Scottsville, Pietermaritzburg 3209, South Africa; shimelish@ukzn.ac.za (H.S.); isackmathew@gmail.com (I.M.); 2 Agricultural Research Council—Small Grain, Bethlehem 9700, South Africa; tsilot@arc.agric.za

**Keywords:** drought-tolerance, genetic resources, selection indices, breeding technologies, *Triticum aestivum* L.

## Abstract

Wheat production and productivity are challenged by recurrent droughts associated with climate change globally. Drought and heat stress resilient cultivars can alleviate yield loss in marginal production agro-ecologies. The ability of some crop genotypes to thrive and yield in drought conditions is attributable to the inherent genetic variation and environmental adaptation, presenting opportunities to develop drought-tolerant varieties. Understanding the underlying genetic, physiological, biochemical, and environmental mechanisms and their interactions is key critical opportunity for drought tolerance improvement. Therefore, the objective of this review is to document the progress, challenges, and opportunities in breeding for drought tolerance in wheat. The paper outlines the following key aspects: (1) challenges associated with breeding for adaptation to drought-prone environments, (2) opportunities such as genetic variation in wheat for drought tolerance, selection methods, the interplay between above-ground phenotypic traits and root attributes in drought adaptation and drought-responsive attributes and (3) approaches, technologies and innovations in drought tolerance breeding. In the end, the paper summarises genetic gains and perspectives in drought tolerance breeding in wheat. The review will serve as baseline information for wheat breeders and agronomists to guide the development and deployment of drought-adapted and high-performing new-generation wheat varieties.

## 1. Introduction

Wheat (*Triticum aestivum* L., 2n = 6x = 42, AABBDD) is a key commodity crop driving food security and the global economy along the value chains. With an increasing world human population and dwindling agricultural lands, the global demand for wheat products will increase by 60% by 2050 [1]. Therefore, wheat yields will need to increase by 1.6% per annum to meet world demands. Recurrent droughts attributable to climate change affect agriculture-based systems through unpredictable rainfall patterns and changes in crop cycles, diseases, and pest dynamics, leading to a reduction in potential yield gains. Consequently, this compromises food security and economic development, among others [2,3].

Low productivity presents a great challenge to wheat farmers due to market fluctuations and price shocks [4]. Hence, there is a need to develop and adopt drought-adapted modern cultivars that are climate-resilient to mitigate the impacts of current and future environmental changes and deliver market-preferred products. Creating drought-tolerant, high yielding and water-use efficient cultivars is the most economical efficient strategy. The use of irrigation water is unaffordable and unsustainable, especially in arid and semi-arid regions, including sub-Saharan African countries. Elucidation of the genetic, agronomic, and environmental components is paramount to determining the response of wheat to drought. Furthermore, the exploitation of genetic resources such as landraces, synthetics and wild relatives will lead to the discovery of a wealth of essential alleles for wheat improvement programs.

Drought stress hampers plant growth, development and yield by changing the inherent agro-physiological and biochemical processes and pathways [5,6]. Drought stress occurs in different patterns and intensities at different crop growth stages [2,7,8]. The impact of drought stress differs according to the genotype, environment and genotype x environment interaction [9]. In addition, drought tolerance is a complex quantitative trait governed by multiple agronomic traits and polygenes [10]. This has posed significant limitations in developing breeding populations and varieties with improved drought tolerance. The temporal and spatial variation in imposing drought stress across different environments and experiments has contributed to poor selection efficiency, slowing genetic progress in drought tolerance breeding. The essential target agronomic traits for improving drought tolerance include early heading, anthesis and maturity [11,12,13,14], as well as root system architecture [15,16]. Physiological traits include relative water content (RWC), canopy temperature (CT), normalized difference vegetative index (NDVI), stem water-soluble carbohydrates (WSC), among others [5,6,17,18,19]. Further, the main biochemical traits associated with drought tolerance include high soluble sugar content, chlorophyll content, reduced gas exchange, high proline content, increased carbohydrates content and reduced superoxide dismutase concentration [6,20,21]. Ideotype breeding with desirable agro-morphological, physiological and biochemical traits can potentially improve drought tolerance in wheat.

Breeding for drought tolerance is an economical approach to increase wheat production and productivity in arid and semi-arid regions [22]. The pace of development of drought-adapted wheat varieties is hindered by several factors, including a lack of robust, high throughput screening techniques, narrow genetic variation, the large genome size (17 gb) of wheat and environmental variance [10,14,23,24]. The success of pre-breeding and creating adequate genetic variability for drought tolerance breeding is affected by (1) the lack of coordinated efforts in exploring and characterising the genetic resources preserved in global genebanks for drought tolerance and (2) the lack of integrated use of genomic and genetic resources and advanced technologies in breeding programs for fast-tracking phenotypic and genotypic selection for drought tolerance [25]. Developments in the state-of-the-art phenotyping and genotyping platforms provide opportunities to enhance selection responses and improve genetic gains in drought tolerance breeding in wheat. Genomic-assisted drought-tolerance breeding has increasingly shown promise, notably in the advent of genotype-by-sequencing (GBS), genome-wide association studies (GWAS), marker–trait association analysis (MTAs) and quantitative trait loci (QTL) mapping. The QTL analyses have increasingly improved the detailed dissection of genes governing complex traits such as drought tolerance and grain yield in wheat [10,26,27]. Additionally, high-throughput molecular markers such as single nucleotide polymorphism (SNPs) are widely used for effective genotyping and marker–trait association. Understanding the underlying genetic, physiological, biochemical, genetic and environmental mechanisms and their interactions are key opportunities for drought tolerance improvement. Therefore, this review aims to document the progress, challenges, and opportunities in breeding for drought tolerance in wheat.

## 2. Impact of Drought Stress on Wheat Production

Drought stress has a catastrophic effect on agricultural production. Table 1 summarises the impact of drought stress on key agro-physiological traits and grain yield in wheat. Agronomic traits such as grain yield were reportedly reduced by 25% to 62.75%, grain numbers per spike by 38% to 50%, 1000-kernel weight by 16.4% to 19.42% and plant height by 14.7% to 34.45% under drought stress across different studies [5,6,28,29,30]. This suggests that grain yield and grain numbers per spike are more sensitive to drought stress. Physiological traits such as leaf water content, photosynthetic rate and chlorophyll content reduced by up to 73.8%, 32% and 19%, respectively, suggesting that leaf water content was more sensitive to drought stress [31,32]. Root biomass and above-ground plant biomass were reduced by 23% and 45%, in that order [33,34]. Hence, there is a need to breed for new varieties that can maintain or partition more biomass under drought stress. A meta-analysis from 144 studies published between 1980 and 2015 showed that wheat yields have declined by 20.6% [2], while a related study that included 60 published studies between 1980 and 2017 showed a reduction of 27.5% due to drought stress at different growth stages [29]. Recently, several countries in Africa experienced drought events which reduced wheat production by 45% [35]. Several factors such as increased population growth, unsustainable agricultural production and ecological imbalance (soil erosion, depletion of nutrients and water resources, overutilization of natural vegetation, and environmental disasters) have aggravated the impact of drought stress in these countries [36].

Drought stress affects wheat at all stages of crop growth. However, its effect is more devastating at the seedling, tillering, jointing, heading, anthesis and grain filling stages [4,29,37]. Drought at the seedling stage can inflict up to a 50% reduction in root length [38]. Notably, reproductive and grain filling stages are the most sensitive stages to water stress [39,40,41,42]. Drought at anthesis causes abortion of ovules, consequently reducing the number of grains per spike and grain weight and ultimately grain yield [5,43,44]. During grain filling stage, moisture deficit may disrupt nutrient uptake and photosynthesis, leading to the production of shrivelled kernels [41]. Bennett et al. [45] reported that drought stress reduced yield by 65%. The authors reported a reduction in the heritability values of grain yield from 74% to 58% due to drought. Plants show a reduced stomatal opening and gaseous exchange when stressed, which leads to low photosynthetic efficiency and yield gains. Thus, drought stress will inhibit trait inheritance, photosynthesis and grain yield.

## 3. Challenges in Breeding for Drought Tolerance

Recurrent drought has become the most prominent cause of reduced yield, grain quality and threat to food security and livelihoods [46]. Challenges in breeding for drought tolerance were reported. There are limited research efforts that identified key root traits in selecting and improving drought-tolerant wheat. Notably, improved root system attributes (e.g., deep and wide-spreading) are desirable for breeding drought-tolerant wheat cultivars [47]. Nevertheless, there is a need to identify key root traits to improve or develop cultivars with improved root attributes and aid in marker-assisted selection. Complementarily, there is a lack of simple and efficient phenotyping methods to improve root attributes as they are labor-intensive and require destructive sampling [48,49]. Developing new high-throughput phenotyping methods that will promote systematic phenotyping of root attributes is of paramount importance. There are limited research efforts that identified key agro-physiological traits in selecting and improving drought-tolerant crops. This is because most drought-adaptive and constitutive traits are controlled by polygenic epistatic and unstable QTL, which are highly influenced by genotype-environment interaction [10,20,50,51,52,53]. This renders low selection efficiency for superior genotypes [54]. Genotype–environment interactions are manifested through crossover ranking and rank inconsistencies when using different indices in identifying drought-tolerant genotypes [55]. Identifying genes associated with drought stress tolerance and their expression and bridging the gap between theoretical research and applied crop breeding is another challenge for brought tolerance breeding [56]. This can be tackled by establishing concerted research groups to reveal the genetic, epigenetic, transcriptomic and metabolomic bases of agro-physiological and root attributes associated with drought tolerance in wheat [57,58,59]. The large genome size (17 Gb) of wheat makes it comparatively more difficult to identify genetic loci controlling key agro-physiological traits conferring drought tolerance in wheat due to its complex genetic background. In this regard, identifying stable QTL or establishing marker–trait associations under contrasting water regimes is crucial for improving drought tolerance using marker-assisted selection (MAS). Extending the genetic analysis research into applied breeding beyond QTL detection has been minimal due to the lack of robust phenotyping and the need for translational genetics. Furthermore, existing mapping populations are routinely used, needing the development of new drought-suited populations. Therefore, new mapping populations sourced from genetically and complementary genotypes will provide avenues for improved drought tolerance [60].

## 4. Opportunities for Drought Tolerance Breeding

### 4.1. Exploring Mechanisms of Drought Tolerance

There are different mechanisms of drought response, including drought escape, drought avoidance and drought tolerance [12,39]. Some of the potential response strategies used by plants to acclimate to drought stress are described in Table 2. Drought escape is an adaptive trait that enables the plants to grow and complete their life cycle before the beginning of severe drought [61]. Early heading, flowering and maturity, reduced plant height and short growth cycle are unique attributes to escape dry spells [11,12,13]. In particular, early heading, flowering and maturity are major drought escape mechanisms that allow the completion of the life cycle before the onset of terminal drought stress, which is common in most rainfed agro-systems [14]. Drought avoidance includes the ability for enhanced uptake of available water and nutrients by a longer or deeper root system [13]. This mechanism is associated with a slow growth rate, small or closed stomata, decreased leaf area, reduced photosynthetic activity and low cell metabolism [61]. Root traits such as increased root biomass, root length density and rooting depth are key drivers of drought avoidance [62]. Decreased leaf area is one of the drought avoidance attributes which results in reduced water loss through transpiration [63]. Drought tolerance is the ability of the plant to maintain its growth, development and reproduction under drought stress conditions [12]. Early maturity and reduced leaf area are common attributes in drought-adapted genotypes. Therefore, understanding plant response to drought tolerance at all growth stages is paramount for breeding. 

### 4.2. Exploring Selection Indices for Drought Tolerance

Use of the target selection and production environments and water stress management remain fundamental approaches in drought tolerance improvement [36,64]. Abdolshahi et al. [48] reported three approaches for breeding drought tolerance. These include (1) breeding for higher yield under non-stress conditions, (2) breeding for maximum yield under drought-prone environments and (3) breeding for drought tolerance using selection indices (traits). For enhanced selection efficiency under non-stress conditions aimed at improving performance under the target drought-prone environment, the procedure assumes the trait(s) measured in two different environments not as one but as two traits correlated genetically. This is because the physiological and genetic mechanisms and the genes required for superior performance may be different under these environments [36]. High genetic correlation of traits under complementary selection environments guarantees higher selection responses for yield and yield influencing traits. Traits with high heritability, genetic advance and genetic gains are essential for direct and indirect selection for better grain yield under different environmental conditions [24,26,79]. Indirect selection involves a selection of one trait via another, while direct selection involves the per se selection of the target trait [36]. The use of integrative traits accompanied by the development and application of new and advanced technologies could accelerate the phenotypic selection of drought adaptive traits and consequently improve yield in marginal/low-yielding environments.

Agronomic traits such as early heading, anthesis, maturity, spike morphology and reduced plant height have been widely targeted in drought tolerance breeding programs [21,42,80,81]. These traits have been used in direct or indirect selection for grain yield and drought tolerance in wheat [21,26,30,42,55]. Drought response varies across the source populations. Hence evaluating each population is necessary for simultaneous improvement of yield and drought tolerance. 

Some physiological traits have been recognised as reliable, cost-effective and non-invasive methods for automated high-throughput phenotyping in crop breeding programs. According to Monneveux et al. [64], physiological traits can be used to select parental genotypes to be used in cross formation. Hence, physiological traits are useful as direct selection criteria for screening populations to eliminate undesirable segregants across generations. Sallam et al. [82] have extensively reviewed drought tolerance-related physiological traits and advances in breeding and genetics research. Key physiological traits are osmotic potential, stay-green, leaf area, relative water content, canopy temperature, normalised difference vegetative index (NDVI), leaf water status and stem water-soluble carbohydrates (WSC) [5,6,17,18]. Stay-green is the ability of a genotype to remain green and continue undertaking photosynthesis due to higher chlorophyll content compared with other genotypes under drought stress [83]. Such genotypes have improved performance under drought conditions with higher grain yield and biomass production [84,85]. SeriM82, a high yielding cultivar released in 1982 exhibited a stay-green phenotype by maintaining green leaf area longer during the grain filling [86]. NDVI is an indirect selection method for stay-green and yield potential [87], while CT denotes the plant’s interaction with the soil and atmosphere whereby plants can mine water under water-restricted conditions [88]. Osmotic adjustments occur when molecular weight accumulates in lower levels of organic solutes [89]. Leaf water status depends on the cell osmotic conditions and water transportation from plant shoots [90]. Canopy temperature [6] and RWC [42] were major yield determinants. Grain yield was associated with CT at both vegetative and grain filling stages [52] and NDVI under rainfed conditions [19]. Furthermore, stem WSC remobilisation during grain filling contributes to grain yield under drought stress [91].

Relatively better yield under drought stress can be achieved by incorporating drought-adaptive biochemical traits from genetically diverse and unrelated parents [30]. Some of the biochemical traits for drought tolerance include soluble sugar content, chlorophyll content, gas exchange, proline content, carbohydrate content, and superoxide dismutase concentration [6,20,21]. Proline content regulates nitrogen accumulation and contributes to membrane stability [92]. Gas exchange is among the key traits susceptible to drought stress. Gas exchange parameters include photosynthetic rate, stomatal conductance, chlorophyll content and water use efficiency [75]. Drought stress tolerance in wheat was associated with high antioxidant enzyme activity, i.e., catalase, glutathione reductase and peroxidase and elevated S-metabolites, i.e., methionine cysteine and glutathione [93,94]. Synthetic derivatives (SYN-DERs) accumulated more soluble sugars, superoxide dismutase concentration, and proline content under drought stress [6]. Proline content was significantly correlated with grain yield suggesting selection efficiency of this trait under drought stress [20]. A reduction in chlorophyll content denotes decreased photosynthesis efficiency [95].

The root and shoot systems are vital for plant growth and development. Exploring genotype plasticity for roots and shoots is useful to improve drought tolerance [26,34,95]. The shoot systems influence plant adaptive response due to differential environmental changes, including drought stress [47]. Roots are primary organs necessary for growth resource acquisition such as water and minerals; thus, wheat varieties with broad environmental adaption and high water use efficiency are the best candidates for breeding. As the soil dries at the surface, a wider and deeper root system ensures access to soil moisture deeper in the profile during water deficit [96]. Thus, breeding for traits such as root length, density, volume, surface area and diameter is an efficient strategy in environments where water deeper in the profile could be available later in the growing season [97,98]. These traits are indirectly involved in water and nutrient acquisition for plant growth [99]. Root length and surface area denote the ability of the plant to acquire soil resources (water and nutrients) [100]. The root length density is used to estimate the soil volume explored by the plant root architecture and consequently the amount of resources (water and nutrients) available to the plant in the soil [97,101]. Root diameter reflects the ability of the plant to adapt to changes in temperature [102], soil texture and water content [103], and mycorrhizal status [104]. This highlights the importance of identifying root system attributes that provide better exploration of the soil profile for resource acquisition and storage, and plant anchorage.

Compared with shallow root genotypes, deep-rooted types have larger-sized grains, higher grain weight and yield. El-Hassouni et al. [105] reported that thousand-kernel weight was 9% higher while grain size and grain yield were 35% higher in deeper rooted genotypes. Root traits can influence stay-green attributes and adaptation to a wide range of climatic or soil conditions [83,106]. Thus, phenotyping of both stay-green and root traits could enable the selection of superior phenotypes for either broad or specific water-stress adaptation.

Plant response or adaptation to drought partially depends on soil properties and the soil water status. Some genotypes respond to drought by maintaining or increasing root growth while decreasing shoot growth [33,107]. Reduced root growth may also occur due to low water status, low oxygen levels (hypoxia or anoxia) and high soil impedance [108]. Early sowing ensures more profound root growth with the ability to access water from deeper soil profiles in drought-prone environments [109]. In this case, increased root versus shoot growth may improve the plant water status under different drought patterns due to enhanced acquisition of water to produce more root tips and maintain the existing shoots. *GmbZIP1* has been linked to increased root and shoot growth under drought [110]. Above-ground biomass was reduced by 45% under drought stress compared to non-stressed conditions in Akron, Ohio, USA [33]. Fletcher and Chenu [111,112] investigated the biomass partitioning of 15 elite Australian cultivars released between 1973 and 2012 and found non-significant changes in the plant biomass and green-leaf biomass at flowering. Only new varieties partitioned more stem and spike biomass at the expense of reduced leaves. Changes in tillering ability have also played a role in the partitioning of stems and spikes. For example, during the 1970s, modern varieties showed increased biomass for every litre of water transpired due to high tillering [112], saving up to 500 g of water per plant up to flowering. According to Kirkegaard et al. [113], a 30 cm increase in root depth into the subsoil could extract an extra 10 mm of water in the deeper soils. *VRN1* is a gene modulating flowering behavior and balance between shoot and root architecture in wheat and barley [114,115]. A spring wheat cultivar Dharwar Dry released in 1994 exhibited superior shoot and root attributes when assessed at different growth stages under multiple growth conditions. This variety can improve root traits in dryland or water-stressed conditions [116]. There have been fewer studies on genetic analysis of root and shoot attributes due to difficulties in phenotyping as they are labour-intensive and require destructive sampling [48,49]. Genetic variation for phenotypic plasticity is threatened by directional selection within a narrow gene pool composed mainly of elite lines. Yet it is imperative to consider the different and divergent sources of genetic variation to develop drought-tolerant and high-yielding varieties. Studies on trait–marker associations and biomass allocation will improve the selection efficiency in conventional breeding programs [26,34].

### 4.3. Genetic Variation as a Source of Drought Tolerance

Genetic variation is the pillar for improving quantitative traits such as yield components and drought tolerance [25]. Table 3 contains some of the sources of drought-tolerant genes reported around the world. Genetic variation can be enhanced via the introduction of existing varieties, developing segregating materials through local or international nurseries, hybridisation and mutation breeding [117]. The extent of natural variability changes with time and space due to evolution, natural selection, artificial selection, mutations, gene flow and genetic drift [16]. The development of cultivars with improved adaptation to biotic and abiotic stresses, including drought stress, hinges on identifying suitable genetic resources with adequate and functional genetic variation for target traits. The use of parental lines of divergent genetic backgrounds, including unrelated and complementary genetic resources possessing suitable drought-adaptive and yield-enhancing traits, ensures the development of superior breeding populations [21,30,67].

Important sources of genes for economic traits in wheat include landraces, elite breeding lines, synthetics and wild relatives [6,118,119]. Among these genetic sources, landraces and drought-adapted varieties are ideal for use in pre-breeding and breeding programs. These genetic resources have high cross-compatibility, wide-adaptation and are rich in farmer- and consumer-preferred traits [25]. Furthermore, improved or breeding lines are essential for the creation of genetic variation with less linkage drag associated with undesirable genes or rare alleles. Some wheat genetic resources such as Dharwar Dry (originated from India), Drysdale, Excalibur and Gladius (from Australia) are widely used in developing genetic populations and drought-tolerant lines [120,121,122,123,124]. The reported genetic resources are divergent in transpiration efficiency, drought tolerance, stay-green and high water use efficiency. The major sources of genetic variation in wheat breeding programs are described below.

#### 4.3.1. Landraces

Landraces are genetically heterogenous breeding stocks adapted to their ecologies and farming systems. They are excellent sources of genes for drought tolerance [65,125]. Aragon 03 was one of the landraces selected for its drought tolerance in the 1940s and is widely cultivated in Spain. This cultivar has exhibited durable drought tolerance and improved traits such as higher pre-anthesis biomass production in different conditions [126]. A Japanese variety, ‘Norin10’ had the *Rht* dwarfing genes (*Rht1* and *Rht2*) [127] while the Aka Komugi landrace harboured the dwarfing *Rht8c* allele [128] that contribute to drought tolerance. Landraces have not been widely used in breeding programs due to a lack of information on their utility and pedigree, limited availability of descriptors, loss of essential alleles due to evolution and domestication processes and the presence of undesirable alleles that may lead to linkage drag. The exploitation of landraces should be prioritized to deliver important alleles/traits in breeding programs, including drought tolerance.

#### 4.3.2. Synthetics

Synthetic hexaploid wheats (SHWs) developed by artificial hybridization between tetraploid wheat (*T. turgidum*) and goatgrass (*A. tauschii*) are valuable sources of drought tolerance genes [129,130]. The major limiting factor in wheat is the narrow genetic variation in the D-genome, thus the SHWs were developed to increase diversity in D-genome for drought tolerance. Useful genes are identified and introgressed via synthetic derivatives or advanced synthetic backcross lines (SBLs). To date, thousands of SHWs have been developed at the International Maize and Wheat Improvement Centre (CIMMYT) using diverse D genome donor species (*Aegilops tauschii*). The D genome of *A. tauschii* has close homology to the D genome of the hexaploid wheat, which increases the chances of transferring polygenic traits. Rosyara et al. [131] conducted a study using CIMMYT’s SHWs to investigate the genomic contribution of chromosome D derived from *T. tauschii.* Their results showed an improved genetic gain for grain yield (25.3%) and maintained higher genetic diversity. CIMMYT’s efforts to widen genetic diversity have produced 1577 SHWs representing 21% of germplasm between 2000 and 2018. Eighty-six varieties were released in 20 countries, with China (34%) having the high adoption rate of these lines, followed by India (7%) [119]. During the past few decades, 30% of yield gains under drought stress were attributed to the use of SHW [132]. Song et al. [130] identified six genotypes that were more drought tolerant than their parents. The genotypes exhibited high antioxidant activities, including superoxide dismutase (SOD), peroxidase (POX) and catalase (CAT) which could have improved drought tolerance under rainfed conditions. These findings demonstrate the importance of SHWs in improving drought tolerance in wheat.

#### 4.3.3. Wild Relatives and Their Progenitors

Wild relatives of wheat and their progenitors, e.g., wild emmer wheat (*T. dicoccoides*) and *Aegilops* (*A. tauschii*) are major sources of drought tolerance genes [133,134,135,136]. Wild-type alleles at the *Rht-B1* and *Rht-D1*, and the presence of the rye translocation (1B.1R) favoured grain yield under drought stress conditions [6]. Wild relative species incurred a lower decline in physiological traits and chlorophyll inflorescence parameters [136]. Like landraces, the major challenge in using wild relatives is the epistatic and pleiotropic effects of some genes associated with rare alleles leading to linkage drag. This can be averted by crossing elite lines to reduce the transfer of rare alleles, simultaneously delivering drought-tolerant genes. This approach will provide avenues for QTL mapping and engineering for drought tolerance in the future.

**Table 3:** Some of the drought-tolerant wheat genetic sources reported globally.

Variety Name	Pedigree	Country/Organisation	Year of Release	References
Katya	Fortunato/No301//Bezostaya 1	Bulgaria	1983	[137]
Mufitbey	Wariquam//Kloka/Pitic2/3/Warimek/Halberd/4/3 ag3 Aroona	TZARI	2006	[137]
Berkut	Irene/Babax//Pastor	CIMMYT	2002	[121,138]
Weebil84	-	CIMMYT	-	[31]
Babax	BOWjNAC//VEEmBJY/COC	CIMMYT	1992	[51,139]
SeriM82	-	CIMMYT	1982	[139]
Pavon F76	VICAM-71//CIANO-67/SIETE-CERROS-66/3/KALYANSONA/BLUEBIRD	CIMMYT	1976	[139]
Opata M85	-	CIMMYT	1985	[139]
Roelfs F2007	-	CIMMYT	2007	[139]
Borlaug100	ROELFS-F-2007/4/BOBWHITE/NEELKANT//CATBIRD/3/CATBIRD/5/FRET-2/TUKURU//FRET-2	CIMMYT	2014	[139]
Sitta	-	CIMMYT	-	[120]
Dharwar Dry	DWR39/C306//HD2189	India	-	[120]
Aragon 03	-	Spain	1940	[126]
Krichauff	Wariquam//Kloka/Pitic2/3/Warimek/Halberd/4/3 ag3 Aroona	Australia	1996	[121]
Excalibur	RAC-l77(Sr26)⁄UNICULM-492⁄⁄RAC-311-S	Australia/University of Adelaide	1991	[122]
Gladius	RAC-875/Kriachauff//Excalibur/Kukri/3/RAC875/Krichauff/4/RAC-875//Excalibur/Kukr	Australia/AGT	2007	[124]

CIMMYT: International Maize and Wheat Improvement Centre. TZARI: Transitional Zone Agricultural Research Institute, Eskisehir. AGT: Australian Grain Technologies.

### 4.4. International Research Collaborations

Breeding for drought and heat stress tolerance was initiated by the International Wheat Improvement Network (IWIN) led by the International Maize and Wheat Improvement Centre (CIMMYT) established in 1966 in Mexico and the International Center for Agricultural Research in the Dry Areas (ICARDA)/Lebanon established in 1977 and partnered with Egypt in 1979. CIMMYT and ICARDA used diverse genetic sources, including landraces, synthetic hexaploid, and wild relatives of wheat such as goatgrass (*A. tauschii*) and durum wheat (*T. turgidum*) in their breeding programs. Both centres adopted complementary breeding technologies involving conventional and molecular breeding approaches to develop high-yielding germplasm with tolerance to heat and drought stress, diseases and insect pests with acceptable end-use qualities [140,141].

Approximately 70% of spring wheat growing areas in developing countries adopted CIMMYT germplasm as either direct release or breeding parents in their new varieties [142]. Moreover, the centre assisted in capacity building through the training of various research experts in global breeding programs. The global coordination of wheat research, human and infrastructure capacity development related to heat and drought stress breeding include the Heat and Drought Wheat Improvement Consortium (HeDWIC), International Spring Wheat Yield Nursery (ISWYN), Semi-arid Wheat Yield Trials (SAWYTs), Elite Spring Wheat Yield Trials (ESWYTs), Wheat Yield Consortium (WYC), and International Wheat Improvement Network (IWIN). These entities are mandated to collectively bring global research expertise and resources through evaluation of wheat under biotic and abiotic stresses in multiple environments, and also to develop new gene combinations with superior varieties, among others. The research consortium has served as a source of new germplasm globally, allowing the use of beneficial gene pools across multiple environments, defined by similar biotic and abiotic stresses and agro-ecologies [143]. For instance, research and development in wheat in South Africa have continued to make use of CIMMYT lines to enhance drought-tolerance improvement and genetic gains through phenotype selection and molecular breeding [10,20,30,144]. The South African Agricultural Research Council-Small Grain (ARC-SG) germplasm bank holds more than 20,000 small grain accessions (mostly imported from global genebanks), including wheat, oats, barley, rye, and tritocosecale, and wheat accounts for 87% of the ARC-SG collections.

### 4.5. Wheat Variety Registration, Deployment and Impacts

Agriculture has been the pillar of human livelihood since antiquity, with the first cultivation of wheat being about 10,000 years ago. Wheat cultivation began using landraces selected based on better yield and quality characteristics. Continuous production, human and natural selection have led to improved varieties with high yields and farmer-preferred traits with high threshability and non-shattering types [145,146].

CIMMYT is actively working with research and development partners of various national governments to improve maize and wheat for enhanced productivity. This includes developing varieties that can adapt to different mega-environments such as high rainfall, irrigated and dryland environments, and high temperature or heat stress-stricken areas [141]. Following this success and initiation of other global breeding programs, hundreds of varieties are continuously being released and distributed worldwide. These varieties are imported and bred with locally adapted varieties to deliver diverse, desirable traits, including biotic and abiotic stress tolerance. 

In the 1940s, the breeding goals of most programs were aimed at yield increase. However, in the 1960s, due to the green revolution, there was a shift in the development of drought-tolerant varieties through breeding local varieties. Following the green revolution initiative, global wheat productivity increased by 3.3% year^−1^ between 1949 and 1978. The yield increase was due to the expansion of production area rather than improved varieties. However, in the 1960s, wheat yield increase was significantly high due to the adoption of improved varieties, use of irrigation, crop protection chemicals and fertilizers. The largest yield gains were mainly recorded in Africa and Asia (productivity gains of 2.6 and 3.5% year^−1^, respectively). Between 1982 and 1991, wheat productivity slowed down to 1.5% year^−1^ except in China, which increased its productivity due to market-oriented reforms in the rural sectors [147].

Table 3 presents some of the drought-tolerant wheat genetic sources reported globally. Aragon 03 was one of the landraces selected from the indigenous landrace population, Catalan de Monte, for its drought tolerance in the 1940s and is widely cultivated in Spain. This cultivar later showed higher biomass under different growth conditions [126]. ‘Norin10’ (released in the 1940s) is a Japanese green revolution variety with dwarfing genes denoted as *Rht1* and *Rht2*, which have semi-dominant genes *Rht-Blb* and *Rht-Dld* [127]. These genes conditioned reduced plant height of 60–110 cm compared to other cultivars with plant height taller than 150 cm [148]. Between the 1960s and 1990s, more than 1500 elite wheat varieties distributed through the international nurseries of wheat breeding were released in different countries [141]. Furthermore, there was excess wheat supply and low food prices during this period due to the impact of the first green revolution in South America and Asian countries [149]. Additionally, there was an increase in income from international export markets, as several countries became net importers of wheat [150].

Genotypes SeriM82 (high yielding) and Babax (drought tolerant) developed by CIMMYT were released in 1982 and 1992, respectively, with relatively high yield performance under drought conditions [151]. Other breeding populations derived from these lines were used in assessing the genetic control of yield and drought adaptive traits under a wide range of environments [51,52,152,153,154]. SeriM82 exhibited a narrow root architecture thus capturing water deeper in the soil [155]. Between 1994 and 2014, public and private breeding programs globally released around 63% and 37% of wheat varieties, in that order. Three CIMMYT-bred varieties such as Roelfs F2007, ONAVAS F2009 and Borlaug 100 were released in 2007, 2009 and 2014, showing significantly high yields of 5.95, 5.83 and 6.58 t/ha, respectively [156]. The release of new varieties helped local and international private seed companies to increasing the adoption of improved varieties by more than 50%. For instance, in Ethiopia, the income gained by wheat farmer’s post-adoption of modern wheat varieties in 2016/2017 was estimated at USD 48 million. This has saved the country a monetary value of USD 65 million that could have been spent on importing wheat [157]. This highlights the potential for wheat variety registration/release and deployment for economic development.

## 5. Breeding Methodologies and Technologies

### 5.1. Breeding Wheat for Drought-Tolerance—Conventional Approaches

Conventional breeding involves the creation of genetic variation through sexual recombination of genes from contrasting parents and selecting superior progenies for developing improved varieties. Different selection methods are used in the conventional breeding of self-pollinated crops, including wheat. These include bulk selection, pedigree breeding, pure line selection, and single seed descent (SSD) selection methods, among others. The most widely used selection methods in wheat improvement are the bulk selection method, pedigree method and SSD [52,153,158,159], which are briefly outlined below.

*Bulk selection* involves hybridisation of parents to produce the F_1_ generation. The F_1_ is selfed to produce the F_2_ generation, and the subsequent generations are harvested in bulk to raise the next generations up to the F_5_ [160]. This method is simple, convenient, cost-effective, and relatively easy and does not require pedigree recording. Line selection is performed at the F_6_ and the evaluations of lines are performed until F_10_ to F_11_ when the population has become homozygous. During this selection method, natural selection is expected to increase the frequency of superior genotypes and improve genotype adaptability to different environmental conditions such as salinity and drought stress [161,162]. The disadvantages of this method include (1) the longer time required to develop a new variety, (2) unavailability of information on trait(s) inheritance since a progeny test is not mandatory, and (3) loss of superior genotypes due to natural selection [160,163].

*Single seed descent* involves advancing the breeding generations through the use of a single seed after the initial crosses are performed. With this method, F_2_ to F_4_ generations are advanced without selection and irrespective of individual plant vigour. Selection is only performed later in the F_5_ or F_6_ when the population is presumably homozygous. This method requires little space, labour and effort and allows rapid advancement of the next generation by retaining sufficiently large and random samples from F_2_ generation. The major advantages of SSD are (1) the homozygosity can be obtained very easily and rapidly (two to three generations per year), (2) it is not affected by natural selection and (3) it is amenable to various selection methods, including speed breeding. The demerits of this method are (1) the selection of plants is based on individual phenotype and not the progeny performance and (2) loss of desirable traits due to selection from a single seed per plant [158].

*Pedigree breeding* begins with the hybridisation of selected pairs of parents, e.g., “a commercial variety and a genotype chosen based on a particular superior trait”. A single plant is selected from the segregating F_2_ population. Progeny performance is evaluated with the repeated selections from F_3_ to F_6_ until the recombined genes are homozygous and the population is homogeneous [158]. This method requires record keeping to track parent–progeny relationships [164]. During pedigree breeding, phenotypic selection is performed in the early generations (F_3–4_), and yield tests are conducted later (F_5_ to F_10_) when the population has reached adequate homozygosity [159]. According to Allard [164], a new variety may be released for commercial production after testing for five years at least at five representative locations.

The success of any phenotypic selection programmes depends on drought-adaptive and constitutive traits that are highly heritable in the breeding generations. Landraces, breeding lines, synthetics, double haploids (DHs) and recombinant inbred lines (RILs) are largely utilised during drought tolerance breeding [25,52,165]. Integration of drought tolerance genes into the high genetic background is often carried out in conventional breeding. For example, a mapping population was developed from a cross of SeriM82 × Babax, whereby SeriM82 was a high yielding genotype while Babax was a drought-tolerant genotype [52]. Conventional breeding requires several generations of screening to identify contrasting breeding parents and develop stable performing varieties through continuous selection across multiple environmental conditions [163]. Contrasting and target production environments are used to identify superior genotypes with specific or broader adaption [6,10,41,51].

Due to the various limitations associated with phenotypic selection, there is the need for complementary breeding approaches such as marker-assisted selection, genomic selection and genome editing to identify and select superior genotypes. This will enable early generation selection and independent to environmental conditions. The advent of molecular markers and genome editing technologies provides opportunities for phenotyping complex traits, thus reducing labour, time and costs for cultivar development. This will improve selection efficiency in conventional breeding, consequently overcoming the shortcomings of conventional breeding.

### 5.2. Breeding Wheat for Drought-Tolerance—Genomic Resources

Genomic resources are routinely used to complement phenotypic selection. DNA markers are used for genetic analysis and identify and select superior genotypes heterotic groups, introduce and track genes in the breeding processes. Marker analysis enable to integrate essential traits and genes conferring the adaptation and performance of wheat genotypes, including drought and heat stress conditions. Marker-assisted selection (MAS) will allow efficient selection irrespective of the stage of the plant growth and without the influence of the environment and thus shortening the breeding cycle [166].

Molecular markers such as single nucleotide polymorphisms (SNPs) and diversity array technology (DArT) have revolutionized the application of MAS in wheat breeding programs [167]. Several markers associated with drought-responsive agronomic traits have been identified [6,10,15,143]. However, the genetic control of drought tolerance is complex due to the large number of genes, unstable QTL, epistatic interaction of QTL and the large genome size and the complicated genetic background of wheat [50]. Furthermore, most marker technologies indicate the presence or absence of a gene without detailed information on its expression and effects on a trait [168].

The advent of genomic resources to avail high-density genome-wide genotype-by sequencing (GBS) have allowed genomic prediction and selection of superior genotypes with multi-genetic traits at the early stages of the breeding cycle [169,170]. In wheat, GBS or next-generation genotyping are valuable tools that are widely used to discover SNPs, identify genetic variations, reduce genome complexity and predict genetic gains [170]. Genomic selection (GS) involves the following steps: (1) phenotyping of diverse sets of populations for different quantitative traits and genotyping across the entire genome to predict the performance of the distinct population, (2) genotyping of breeding population to estimate the genomic estimated breeding values (GEBVs), (3) validation of both sets of populations by phenotyping and genotyping and (4) investigation of genetic gains over time [141,170,171]. GS is carried out to accommodate all minor-effect QTL, to identify individuals with the highest GEBVs for target traits and to reduce the number of generations required to select a superior phenotype. The combination of GS with phenotypic selection (PS) showed an improvement of yield by 23%, indicating that genetic gains could be improved by complementing conventional PS with GS in breeding programs [170]. However, GS is yet to be explored for drought tolerance breeding since drought patterns may vary over space and time across wheat varieties and locations.

### 5.3. Gene editing Technologies and Breeding for Drought Tolerance

The wheat genome is a complex build-up of genes from three genomes (AABBDD, 2n = 6x = 42) [172]. This renders complex regulatory pathways to interact, constitute and maintain genetic homeostasis. Genome editing alters particular genomic regions through insertion, deletion and substitution of genes [172,173,174,175,176]. Thus, any new gene(s) inserted or substituted in the genome should be stable and in a desirable direction [177].

In the past 10 years, genome editing has enabled scientists to generate targeted modifications in organisms of interest [178,179]. The genetic engineering approach efficiency in organisms through genome editing involves nucleases: zinc-finger nucleases (ZFNs), transcription activator-like-effector nucleases (TALENs) and the clustered regularly interspaced short palindromic repeat (CRISPR/Cas systems) [179,180] such as CRISPR/Cas9, CRISPR/sgRNA and CRISPR/Cpf1 [181,182,183,184]. Notably, CRISPR/Cas9 could aid in the rapid improvement of drought and locally adapted varieties by integrating genes from wild relatives showing drought tolerance. This will deliver new commercial varieties that still retain stress resistance traits of their wild relatives [185].

The use of genomic resources and technologies could accelerate molecular breeding and improvement of crops for adaptability to abiotic and biotic stresses. Intra-or inter-genus and species transfer of alien genes for drought and heat tolerance can be deployed successfully to improve wheat adaptability. For example, the transfer of foreign genes modulating stress-adaptive traits, including hormones, dehydration-responsive element-binding proteins (*DREB*), enzymes and deeper rooting genes (*DRO1*) have proved that wheat adaptation and performance can be improved [186]. An example of intragenus transfer of a gene(s) includes *DRO1* and *DREB* genes. *DRO1* controls root growth angle in rice [82,187] and influences the orientation of the root system in wheat [186]. *DREB* are involved in tolerance to numerous abiotic stresses, including drought, salinity, low temperature and abscisic acid (ABA) in wheat [188], and drought tolerance and growth retardation in rice [189].

The application of these technologies is not limited to drought tolerance studies. For example, CRISPR/Cas9 technologies have confirmed their simplicity, proficiency, flexibility and wide adaptability and applicability in several plant-based applications [190]. For example, they have been used for targeted mutagenesis in chickpeas to unravel responsive genes under drought stress [191]. Loss-of-function mutations are the most genomic modification that occurred during the domestication whereby they were stacked in key genes controlling traits such as flowering, seed shattering, colour and size through the application of CRISPR/Cas9 [192]. This has enabled breeders to retrace thousands of years of crop improvement in the process of de novo domestication.

One of the main limiting factors to genome editing is the plant transformation efficiency, which hinders the transfer of edited material into the target cells [185]. The role of genome editing is to change an organism’s DNA (add, remove or alter) at a particular location in the genome. Therefore, repairing the pathways of DNA double-strand break (DSB) are homology-dependent repair (HDR) in which a donor sequence matching the target is copied, and nonhomologous end-joining (NHEJ) in which rejoining the broken ends can lead to mutations at the break site [175]. The advent and high potential of genome editing in crops are continuing to drive the development of more effective plant transformation approaches.

### 5.4. Biotechnological Approaches

#### Molecular Markers, Genetic Engineering and Associated Technologies

Biotechnological approaches such as omics and genetic engineering (GE) technologies have been incorporated into breeding programs for accelerated breeding and increase genetic gains in drought tolerance [193]. Table 4 presents the application of different biotechnology approaches to various crops globally. Transgenic and cisgenic are two GE approaches that have advanced the integration of high value traits [194,195]. Transgenic involves transfer of desirable genes derived from target organisms through vector and tissue culture systems or particle bombardment [196]. Cisgenic is termed as the use of recombinant DNA technology to transfer genes from complementary parents (crossable and sexually compatible species) without altering much of the genetic background of the recipient parent [194]. Cisgenesis is almost similar to conventional breeding as it combines the phenomenon with modern biotechnology approaches. Cisgenesis has more impact when used (1) on traits that are dominantly inherited and (2) when applied in translocation or introgression breeding [197]. The two GE approaches have significantly advanced the improvement of targeted traits in wheat. For example, the expression or overexpression of genes *GmDREB* and *TaPEPKR2* improves drought tolerance [198,199]. Overexpression of *TaNAC69* has significantly increased root biomass and longer and deeper roots in bread wheat [200], while the transfer of *1Dy10* has improved end-user quality traits such as bread baking quality in durum wheat [201].

The release of genetically modified (GM) crops is regulated to prevent potential negative and harmful effects to human health and the environment. These regulations are based on transgenic organisms (non-crossable species) and cisgenic organisms (crossable species). However, cisgenesis as an advancement of traditional breeding can be effective if excused from GM regulation [202]. This is because, in GM application, the gene source is more prioritised than the technology of gene transfer [197]. In 2007, GM crops were grown on 114 million hectares and increased to more than 179 million hectares in 2015 (more than 10% of world’s arable land) globally. GM soybean and maize for drought tolerance have been developed and commercialized for production; however, progress in the development of GM wheat is still an infant. For example, in Africa, GM are grown in three countries: South Africa (cotton, maize and soybean (2.3 million ha, 19.13% of Africa’s share)), Burkino Faso (cotton (0.4 mha, 7.02%)) and Sudan (cotton, 0.1 mha, 0.41%), with the common crop being GM cotton [203]. Currently there is no GM wheat commercialised anywhere globally. Khan et al. [204] comprehensively reviewed the progress in developing drought-tolerant transgenic wheat and reported that there is no drought-tolerant transgenic wheat approved for commercialisation. This is because most evaluated transgenic wheat lines fail to perform under stressed environments [205,206].

Marker technologies such as marker-assisted back cross (MABC) breeding enable the transfer of targeted genes/QTL at target loci from complementary parents with two or three backcrosses. MABC is presumably one of the convenient and cost-effective forms of marker-assisted selection (MAS) involving the transfer of targeted traits from a donor parent to a recurrent elite parent without considerable change in the genetic background of the recurrent/recipient parent [207]. This approach is efficient on crops with traits of low heritability values that is difficult to phenotype and select using key quantitative traits, especially those expressed later in the growth stage [194]. However, transferring one gene at a time using MABC can be a challenge, given that quantitative traits are affected by environmental conditions and epistatic and polygenes [25]. Though the MABC approach has enabled accelerated improvement of drought tolerance in wheat [208,209], successful MABC studies for drought tolerance are still marginal.

**Table 4:** The application of biotechnology approaches on various crops globally.

Crop	Gene Expression	Gene	Trait Descriptions	References
**Intragenesis/Transgenesis**				
Wheat	Overexpression	*TaNF-YB4*	Produces more spikes and increases grain yield.	[124]
Wheat	Expression	*GmDREB*	Confers drought tolerance, produces more leaves, roots and high soluble sugar contents.	[198]
Wheat	Expression	*HVA1*	Improves biomass and water use efficiency.	[210]
Wheat	Overexpression	*AtHDG11*	High grain yield and induce changes on physio-morphological traits such as higher proline content and photosynthesis, lower stomatal density, lower rate of water loss, and increased activities of catalase and superoxide dismutase.	[211]
Wheat	Overexpression	Ferritin gene, *TaFER-5B*	Improves leaf iron content and ROS, confers tolerance to drought and temperature.	[212]
Wheat	Overexpression	*TaNAC69*	Increases root biomass and longer and deeper roots.	[200]
Wheat	Expression	*PEPC*	Higher proline, soluble sugar and water use efficiency, more extensive root system as well as increased photosynthetic capacity.	[213]
Wheat	Expression	*HaHB4*	Increases yield and water use efficiency.	[214]
Wheat	Overexpression	*CspA and CspB*	Lower rate of water loss and MDA content, higher chlorophyll, proline and grain yield.	[215]
Wheat	Overexpression	*TaPEPKR2*	Enhances drought tolerance, higher root length.	[199]
Wheat	Expression	*TaSnRK2.8*	Enhances tolerance to drought, salt and cold stress. Other traits include longer primary roots and various physiological traits, including higher relative water content, strengthened cell membrane stability, significantly lower osmotic potential, more chlorophyll content,	[216]
Wheat	Overexpression	*TabZIP2*	Reduces spikes and seeds, increases single seed weight.	[217]
Potato	Silencing	*GBSS*	High amylopectin.	[218]
Potato	Silencing	*StAs1*, *StAs2*	Limits acrylamide in French fries.	[219,220]
Potato	Silencing	*Ppo*, *R1*, *PhL*	Prevents black spot bruise, limit cold-induced degradation of starch and limits acrylamide in French fries.	[220,221,222]
Apple	Expression	*HcrVf1*, *HcrVf2*	Resistance to scab.	[223]
Strawberry	Overexpression	*PGIP*	Resistance to grey mould.	[224]
Alfalfa	Silencing	*Comt*	Reduced lignin levels.	[225]
**Cisgenesis**				
Durum wheat	Expression	*1Dy10*	Improves end-user quality traits such as bread baking quality.	[201]
Barley	Overexpression	*HvPAPhy_a*	Improves grain phytase activity.	[226]
Grapevine	Expression	*VVTL-1*, *NtpII*	Resistance to fungal disease.	[227]
Potato	Expression	*R-genes*	Resistance to late blight.	[228]
Apple	Expression	*HcrVf2*	Resistance to scab.	[229]
Apple	Expression	*Rvi6*	Resistance to scab.	[230]
Poplar	Overexpression	*Growth genes*, *PAT*	Different growth types (rate of regeneration of transgenic shoots, growth rate, plant size and architecture).	[231]

### 5.5. Genes Associated with Drought Tolerance

Several studies have reported genes linked to important drought-responsive traits (Table 5). A gene is considered a candidate when it is associated with a known or proposed function determining the QTL of a trait of interest. As drought brings about changes in gene expression, identification of the expression of candidate genes under drought stress is important. Wheat genotypes use multiple mechanisms to respond to drought stress and numerous genes condition these mechanisms. This includes genes involved in the coding proteins involved in osmotic adjustment, repairs, transcriptions and regulations [134,232,233,234,235]. Several bio-chemicals assist in regulating dehydration membrane stabilization and osmotic adjustments, among other functions [50,236], thus enabling wheat response to drought at different growth stages [57].

Genes controlling root architecture play a significant role in resource acquisition such as water and nutrients and have been widely targeted in drought tolerance breeding. DEEPER ROOTING1 (*DRO1*) gene, a rice QTL controlling root growth angle reportedly played a significant role in altering root system architecture, thus improving drought avoidance [186,253]. Rice *DRO1* orthologs and wheat *DRO1* orthologs share 76% identity, suggesting the possibility of functional similarity and potential contribution in manipulating root surface area (RSA) for drought avoidance in wheat [235]. Deep-rooted plants contribute to drought avoidance by extracting moisture from deeper soil layers [13]. A field study assessed the effect of drought on changes in wheat transcriptome during the early reproductive stage and discovered 309 differentially expressed genes (*DEGs*) involved in various critical processes such as floral development, photosynthetic activity and stomatal movement [57]. Candidate gene *TaELF3* for earliness per se (Eps) locus has proved to play a significant role in regulating flowering time [254]. Green revolution genes such as *Rht-Blb* and *Rht-D1b* significantly reduce plant height in wheat [255]. *GPC-B1* is an important gene regulating gluten protein content in wheat, thus significantly affecting grain yield [165]. Genes such as *TaSNAC8-6A*, *TaMYB3R1* and *TaNAC69* have been reported to contribute to drought response in wheat [200,246,252]. This represents valuable wheat genetic resources for the improvement of drought tolerance. However, the impact of these genes on grain yield remains to be elucidated. Gene pyramiding and stacking by crossing complementary drought tolerance genotypes/traits from different growth stages could boost drought adaptation and grain yield.

Gene cloning in wheat has been achieved using comparative genomics approaches between wheat and rice, resulting in yield-related genes such as *TaTGW6* [256], *TaGW2*-*6A* [257] and *TaGS*-*D1* [258] and *TaSus2* [259] among others. *TaMYBsm3* and *TaCRT1* are among other genes cloned for wheat adaptation to abiotic stresses such as drought [239,250]. The discovery of these genes has provided useful information in understanding the genetics of wheat adaptation to target environments, yield stability, and its contributing traits’ performance under such conditions. Such breakthroughs can be utilised in MAS and genomic selection to accelerate breeding, variety development and deployment.

### 5.6. QTLs Associated with Root and Shoot Attributes under Drought Conditions

Genetic improvement of drought-responsive root attributes and their contribution to higher and stable grain yield through the integration of advanced genomic approaches is of immense importance. Initially, QTL mapping involved the use of bi-parental crosses in different genetic backgrounds. This approach enabled estimation of the number of genomic regions controlling the specific traits in defined populations, characterization of the genomic regions with regard to map position, gene function, phenotypic and pleiotropic effects and epistatic interactions with other QTL. However, this was limited in allelic diversity, genomic resolution and the longer time required to develop mapping populations.

Genome-wide association study (GWAS) and/or marker–trait association (MTA) approach overcomes several QTL mapping limitations by producing higher resolution, based on linkage disequilibrium across the genome, exploiting/employing data from diverse genetic backgrounds, making these approaches more efficient [81,143,260,261]. With GWAS/MTAs, an extensive collection of wheat germplasm is genotyped with SNPs or DArT markers throughout the genome to identify associations with the phenotypic trait(s) of interest [49,143,262,263,264]. However, the large and complex wheat genome, and incompatible genome sequence make GWAS and MTAs studies challenging for identifying genomic regions underlying the observed phenotypes. The availability of wheat sequence reference genome has allowed annotation of functional genes [265], thus enhancing understanding of genome architecture, gene expression, the relationship between drought tolerance genes/QTLs and their conditioning factors [262,263,264,266]. In the past 68 years (1947–2015), multi-trait MTAs or genomic regions have significantly contributed to yield gains of 2.63 to 25.7 million tons [262]. Nearly 800 MTAs/GWAS were reported for drought-responsive traits, i.e., agronomic, physiological, roots and its related traits [234]. Significant MTAs were reported for 36 agro-morphological traits [81].

Mwadzingeni et al. [267] have comprehensively documented genomic regions associated with agro-morphological traits. A summary of recent efforts in QTL and association mapping for important root and shoot traits associated with drought tolerance is presented in Table 6. However, genomic studies on the relationship between root and shoot as well as yield traits remain to be elucidated. This is because root and shoot traits are complex, controlled by polygenes, QTL and environmental effects; therefore, it is challenging to quantify under field conditions. Despite the likely importance of roots and shoots in wheat performance and drought tolerance, few genomic studies on these traits have been undertaken [26,49,266]. Wheat genome B and D [268] have shown to be the main genomes influencing root traits response to drought stress suggesting that these regions are pleitropic and have multiple genes influencing root development. Wheat genome D contained the most loci for root traits under drought-stressed conditions [15,49]. Kabir et al. [269] found chromosomes 2A, 3A, 4D and 5A in a DH population and chromosomes 3B, 4A, 4D and 5B in RIL population as the main loci influencing root parameters. Chromosome 4D harbours pleiotropic QTL for root traits in DH and RIL populations, while chromosome 3A had the pleiotropic markers in the DH population. In addition to locus 3A, chromosomes 2B and 2D have shown pleitropic QTL for root parameters [270]. QTL on 1B, 3D, 4D, 5A and 5B for coleoptile length were found while chromosome 3D, 4D and 5A showed pleiotropism for plant height [271]. QTL for coleoptile length were found on chromosomes 3B and 4B [272]. These results show that QTLs for root parameters are genetically complex and highly influenced by the growth medium and the plant genotype. Some favourable alleles are not well recorded from different environments and genetic backgrounds, thus presenting opportunities in deploying specific alleles with the use of molecular markers. Therefore, it is important to develop accurate, reliable and well-defined phenotyping assays and techniques to elucidate the mechanisms underlying tolerance to drought stress and high yield under drought-stressed conditions. Identifying genomic regions associated with important breeding traits under rainfed and drought stress conditions and characterization of their genetic make-up is of paramount importance. This will improve the development of breeding pools with positive and beneficial alleles and introgression through MAS. Current and future breeding programs can devise strategies to accumulate these alleles to increase genetic gains.

## 6. Progress in Breeding for Drought Tolerance in Wheat

### Breeding Progress and Genetic Gains

Breeding progress under drought-free environments has shown an ever-increasing yield trend, and selection based on this may not give adequate results for drought-tolerant varieties [36,277]. This is because it is practically impossible to combine traits/genes responsible for superior performance in all environments into a single variety. Furthermore, drought is a moving target that cannot be addressed through breeding for neither specific nor wide adaptation. On the contrary, progress in breeding and genetic gains under water-limited environments have slowed down over the years due to variable climatic conditions from year to year, lack of breakthrough germplasms, inefficient breeding strategies and challenges in identifying key breeding traits as well as large genotype-by-environment interaction [36,278]. Though, selecting under “intermediate” environments can be a good alternative over either selection at high or low yielding environments, there is no straightforward criterion to determine the intermediateness of a distinct environment. Selection of varieties under both high and low yielding environments could be one of the sustainable approaches to develop varieties suited to both conditions.

To sustain the current and the future wheat demands, it is imperative to assess the genetic gains and their impact on breeding programs. Determining breeding progress and the rate of genetic gains is important for improving the selection efficiency. This considers the effect of the genotype, environment and their interaction. Phenotypic data from representative germplasm samples evaluated across years (historical data) or altogether in an experiment (era trials) is used to realise genetic gains. CIMMYT has played a significant role in breeding and global distribution of wheat varieties adapted to marginal environments for evaluation under multiple environments and collaboration with national breeding programmes (Table 7) [279,280]. Majority of the documented yield progress under marginal environments has been due to testing under several biotic and abiotic stresses including drought stress. Furthermore, much of the reported yield increase was on the basis of selection under optimum conditions. Nevertheless, the rates of genetic gain are still marginal to meet the projected wheat demand by 2050 [22] needing dedicated wheat breeding programs for heat and drought stress tolerance.

Though CIMMYT data represent international yield trends, national yield trends are a prerequisite to serving as a guide for searching for new innovations and germplasm sources. For example, more than 25,000 yield observations from 26 wheat varieties released by the Agricultural Research Council in South Africa to the world from 1992 to 2012 led to countrywide genetic gains of 0.75%, 0.30% and 0.093% for winter, facultative and irrigated spring wheat [3]. Strategies such as high-throughput phenotyping (HTP), use of improved cultivars, genomic selection and integrating traits through crossing at existing loci guided by MAS could greatly increase genetic gains in wheat [286]. Crop management practices such as irrigation expansion, use of insecticides and fertilisers could also contribute greatly to yield increase.

The major limiting factor for breeding progress and genetic gains is the reduced genetic diversity. Over the last few decades, there have been concerns about increased crop uniformity and reduced genetic diversity [287]. Some assessments revealed inconsistency contributed by different plant breeding methods perceived to reduce genetic gains. Van de Wouw [288] asserted that genetic erosion occurred through replacement of landraces with modern varieties and modern breeding approaches or practices. The use of genetic resources such as landraces, obsolete lines and modern pure or breeding lines have potential to contribute to increased genetic gains.

## 7. Outlook and Conclusions

Wheat breeders and agronomists strive to increase rates of genetic gains for grain yield to support a growing population. Though yield improvement is a key target for most breeding programs globally, current breeding pipelines are not optimised for selecting drought adaptive and constitutive traits and yield. Selection accuracy for quantitative traits may be accelerated by evaluating more selection candidates in multi-environments using well-defined phenotyping assays and phenotyping tools to deliver drought-tolerant varieties. Various selection methods for grain yield and its contributing agro-morphological traits under drought conditions, molecular markers, and genomic regions have been adopted to develop drought-suited varieties. However, simultaneous improvement of drought tolerance and yield through its breeding traits has been perceived as challenging due to inadequate phenotyping techniques, challenges in identifying key breeding traits and large genotype by environment interaction. The large and complex genome size of wheat influencing the expression of multiple genes and QTLs under a given set of conditions makes breeding for quantitative traits complicated. Labour costs, time and space constraints are among major limiting factors.

Strategies such as the cultivation of high-yielding cultivars, high-throughput phenotyping, increased irrigation and water use efficiency are of immense importance. Understanding of environmental variables and agronomic factors determining wheat response to drought and yield is paramount. The exploitation of genetic resources such as landraces, synthetics and wild relatives will lead to the discovery of the wealth of important alleles that can be used in breeding and improvement programs. Advances in molecular markers and marker technologies such as QTL analysis and detection could accelerate genetic selections for significant breeding traits thus reducing the breeding cycle. The availability of wheat sequence reference genome has allowed annotation of functional genes, thus enhancing discovery and understanding of genome architecture, gene expression, the relationship between drought tolerance genes/QTLs and their conditioning factors. Improvement in data analysis techniques will give more power to identify genes and trait associations as well as guide breeders on the yearly status or progress of breeding in their programs. Therefore, elucidation of genetic loci underlying significant breeding traits facilitating wheat adaptation and tolerance to drought and their expression patterns to drought stress will provide a strong foundation for knowledge-based breeding approaches and strategies for improved germplasm for multi-environment and environment-specific niches.

## Figures and Tables

**Table 1:** Impact of drought stress on agro-physiological traits in wheat.

Agronomic Trait	Reduction (%)	Location/Country	References
Plant height Number of tillers per plant Grains per spike Grain yield 1000-grain weight	34.45% 25.43% 38.10% 62.75% 19.42%	Pakistan	[6]
Biomass Grain yield	27.05% 25%	China	[29]
Grain numbers per spike Individual grain weight [mg] Leaf photosynthetic rate Chlorophyll content Spikelet fertility	48% 35% 32% 19% 29%	Kansas State University, USA	[32]
Plant height Days to 50% heading Number of effective tillers Spike length 1000-grain weight Grain yield Biomass Harvest index	14.7% 4.78 36.3% 23.7% 16.4% 43.2% 32.9% 12.7%	Egypt	[5]
Number of grains per spike	50%	South Africa	[30]
Root bimass	23%	South Africa	[34]
Grain yield	40%	South Africa	[28]
Above-ground biomass	45%	Colorado State University, USA	[33]
Leaf water content (LWC) in cultivars Seri M82 and Weebil4, respectively	64.9% 73.8%	Philippines	[31]
LWC in cultivars Kukri and Excalibur, respectively	72.6–54.4% 74.5–50.5%	Australia	[17]

**Table 2:** Agro-morphological traits responsive to drought stress in plants.

Trait	Function in Plants	References
Early growth	Reduces moisture evaporation from the soil surface and increases soil water available for transpiration and growth	[64]
Root system architecture (RSA)	Plays a vital role in the growth, development and overall productivity of the plants	[65]
Long and thick stem internodes	Plays a vital role in the storage of carbon products	[64]
Long coleoptiles	Favoured by deep sowing, functions to avoid extreme hot temperatures from the soil surface, and avoid soil drying. Covers the emerging shoot or first leaf during germination	[64,66]
Tiller numbers	Determines the development of reproductive organs (spike, spikelets, and florets)	[4]
Heading and anthesis	Improve the translocation of assimilates	[67,68]
Longer grain filling	Associated with drought tolerance	[69]
Spike photosynthetic capacity	Contributes to remobilization during grain filling	[64]
Reduced plant height	Associated with resistance to lodging, reduce the moisture demand and prevent moisture loss due to transpiration	[70]
Stomatal conductance	Increased water intake	[71]
Presence of awns	Contribute to photosynthesis and efficient water use. Influences spike length, increases grain size and grain yield under drought stress	[72,73]
Cell membrane stability	Enables continuous leaf functioning at high temperature	[74]
Delayed leaf senescence	Influences grain yield	[75]
Canopy temperature	Enables plants to extract moisture from deeper soil profiles	[76]
Leaf rolling	Helps plants acclimate to moisture deficit	[77]
Chlorophyll content	Specifies a plant’s photosynthetic capacity and accelerates plant productivity, and plant physiological and phenological status	[78]
Large grain size	Emergence, early groundcover, initial biomass	[64]

**Table 5:** Putative genes associated with target agro-physiological traits conditioning drought tolerance.

Gene	Crop	Function	References
*Dreb1*	Wheat	Drought tolerance	[237]
*TaERF3*	Wheat	Drought and salt tolerance Drought avoidance	[238]
*VRN1*	Wheat and barley	Regulates flowering behaviour Balance between shoot and root architecture Modulate plant morphology	[114,115]
*TaCRT1*	Wheat	Calreticulin Ca2+ binding protein	[239]
*Ppd-D1* and *Ppd-D1a*	Wheat	Flowering time determinant gene, involved drought tolerance Photoperiod insensitive allele	[6]
*TaZFP22*, *TaZFP34*, and *TaZFP46*	Wheat	Root expressed and drought induced Q-type C2H2 zinc finger transcriptional repressors in wheat	[240]
*TaER1 and TaER2*	Wheat	High transpiration efficiency and grain yield	[241]
*TaNAC69*	Wheat	Photoperiod insensitivity	[200]
*TaWRKY 1*	Wheat	Confer drought tolerance	[242]
*TaWRKY44*	Wheat	Confer tolerance to multiple abiotic stresses such as drought, salt, and osmotic stress	[243]
*TaSnRK2*	Wheat	Encodes sucrose non-fermenting 1-related protein kinase and adapt to various environmental conditions with significant correlation to spike length and thousand kernel weight	[244]
*TaH2B-7D*	Wheat	Confer drought tolerance, increases relative electrolyte leakage rate and malonaldehyde (MDA) content	[245]
*TaWRKY8*	Wheat	Grain yield and abiotic stress tolerance	[53]
*TaMYB3R1*	Wheat	Salt, vernalisation and drought tolerance in wheat	[246]
*TaAQP7*	Wheat	Drought tolerance in Arabidopsis	[247]
*SNAC1*	Wheat	Salt and water stress tolerance in transgenic wheat	[248]
*GmbZIP1*	Wheat	Drought tolerance	[110]
*TaSST-D1* and *TaSST-A1*	Wheat	Water-soluble carbohydrates, Increased thousand-kernel weight (TKW), plant height and drought adaptability	[249]
*TaMYBsm3*	Wheat	Drought adaptation	[250]
*TaEXPA2*	Wheat	Confer drought tolerance	[251]
*TaSNAC8-6A*	Wheat	Drought adaptation	[252]

**Table 6:** Putative QTL regions for drought-related traits in wheat mapping populations under either individual or drought, heat and non-stressed conditions.

Chromosomes	Associated Roots and Related Trait[s]	Study Approach	Collection or Population Type	References
1D, 2A, 2B, 2D, 3A, 4A, 4B, 5A, 5B, 5D, 6D, 7A, 7D	Average root diameter, number of root crossing, number of root forks, number of root tips, root volume, surface root areas	QTL mapping	Advanced backcross population	[15]
1A, 2B, 3A, 3D, 4B, 4D, 6A, 6B, 6D, 7B	Maximum root length, primary root length, lateral root length, root tip number, total root length	QTL mapping	RIL population	[16]
2A, 2B, 3A, 4B, 4D, 5A, 6A, 6D, 7B	Total root length, total root surface area, total root volume, number of root tips, main root length	QTL mapping	DH population	[269]
1B, 2B, 3B, 4A, 4D, 5A, 5B, 7A			RIL population	
3B, 4D	Root re-growth, root tolerance index, aluminium tolerance	QTL mapping	D-genome substitution lines	[273]
5A	Stay green	QTL mapping	DH population	[19]
1A, 1B, 2A, 3A, 6A, 6B	Root length, root volume, root surface area, number of tips	QTL mapping	RIL population	[274]
2B, 2D, 3B, 3D, 4B, 4A, 6D, 7B, 7D	Maximum root length, root fresh weight, ratio of root water loss, total root length, total root surface area, total root volume, number of root tips, number of root forks	QTL mapping	RIL population	[269]
1A, 1B, 2B, 2D, 3A, 3B, 5A, 5B, 6A, 7A	Maximum root length, seminal root number, total root length, project root length, root surface area, seminal root angle, grain yield	QTL mapping	DH population	[268]
1B, 2A, 2B, 2D, 3D, 4A, 4B, 5A, 5B, 6A, 6B, 6D, 7A	Root depth at booting and mid-grain fill stage, root dry weight at booting and mid-grain fill stage	GWAS	Core collection	[266]
2A, 2B, 2D, 5B	Total root length, root fresh weight, maximum root length, nodal roots, root density, root diameter	MTA	Core collection	[275]
1B, 2A, 2B, 3B, 5A, 5B, 6A, 7A	Total root number, root dry weight, seminal root angle, seed weight, seed length	GWAS	Landraces	[276]

DH: Doubled haploid; MTA: Marker–trait association; QTL: Quantitative trait loci; RIL: Recombinant inbred line.

**Table 7:** Rates of yield gains realised from international breeding programs using CYMMIT lines under marginal environments.

Years	Rate of Yield Increase	Target Environment	References
2007–2016	0.93% (40 kg ha^−1^ yr^−1^)	Drought prone	[281]
2006–2014	2.7% (88 kg ha^−1^ yr^−1^)	Drought prone	[282]
2002–2014	1.8% (0.15–3.5 t ha^−1^ yr^−1^)	Drought prone	[283]
1965–2014	17.7 to 25.6 kg^−1^	Drought prone	[139]
1994–2010	0.7% (2.07–2.7 t ha^−1^ yr^−1^)	Drought prone	[280]
1991–1997	0.09% (2.1 kg ha^−1^ yr^−1^)	Low yielding environments	[284]
1979–1999	3.48% (2.3 to 3.5 t ha^−1^ yr^−1^)	Drought prone	[285]
1979–1998	0.19% (5.3 kg ha^−1^ yr^−1^)	Drought prone	[284]
1979–1995	2.75% (70.5 kg ha^−1^ yr^−1^)	Drought prone	[285]
1977–2008	0.5% (251 to 291 g m^−1^ yr^−1^)	Drought prone	[279]
1964–1978	1.54% (2.3 to 4.3 t ha^−1^ yr^−1^)	Drought prone	[285]

## Data Availability

Not applicable.
